# Editorial Announcement

**Published:** 1913-04

**Authors:** 


### Editorial Announcement.

As the Abstracts are the only part of the Journal which can be cut off at any point to complete a page and left over—not too long—without detriment, and as all other departments necessarily vary in space from issue to issue, it has been decided to place them at the end of the Journal, except such as are required to complete pages in the Original Department, and thus allow every original article to begin at the top of a page. This will save at least a day's time in printing.

Our readers may have noticed that the Department of Miscellany has been discontinued, items that might be placed in it, having been relegated to the advertising pages, topics of interest, etc., or omitted as not appropriate to a medical journal. We have increased the average issue by four pages. More important has been the effort to condense in all departments, so far as practicable, and to omit meditative articles and addresses from the Original Department.

We ask the opinion of our readers on two points: Shall we continue the Department of New Inventions or shall it be discontinued or greatly reduced by selection? Shall the Personals be maintained as at present, or shall they be reduced to voluntary notices of removal, extensive travel, etc.? It is scarcely necessary to add that we always welcome suggestions as to means of improving the JOURNAL—which include $ome nece$$itie$ which are obviou$.

---

Regarding some suggestions that we establish a bureau of advice regarding problems arising in practice, or that the regular editorial section include articles on analysis of urine, stomach contents, therapeutic notes, etc., we want, first, to express our thanks and to make the following explanation. As to the bureau of questions and answers, we know just about enough to dig out a diagnosis and prescribe the proper treatment, in a moderate percentage of cases and in a rather limited field, when the patient is on hand for physical examination and every facility is afforded for a careful personal study. To give advice on second-hand information, and in an off-hand way, on all sorts of conditions, is beyond the power of the editor. It has seemed wise, also, to avoid entering the field of original scientific articles and not to attempt dictactic discussions drawn largely from textbooks that might better be followed directly by the reader.

